# A diagnostic approach for defining idiopathic remitting diabetes: a retrospective cohort study

**DOI:** 10.1186/1472-6823-14-45

**Published:** 2014-06-09

**Authors:** Tarig Babiker, Ali J Chakera, Maggie Shepherd, Andrew T Hattersley

**Affiliations:** 1 Department of Diabetes and Endocrinology, Royal Devon and Exeter NHS Foundation Trust, Exeter, UK; 2 NIHR Exeter Clinical Research Facility, University of Exeter Medical School, Barrack Road, Exeter EX2 5DW, UK

**Keywords:** Diabetes, Remission, Criteria, Diagnosis, Remitting diabetes

## Abstract

**Background:**

11 patients were referred to our Molecular Genetics Department at the Royal Devon and Exeter Hospital between 2000-2012 with a physician’s diagnosis of remitting diabetes. Our aim was to identify patients with remitting diabetes whose clinical presentation is not explained by any known aetiology of diabetes.

**Methods:**

We obtained longitudinal clinical data on all 11 patients from the hospital records. All patients were aged between 0.5 and 35 years at diagnosis. We applied clinical criteria derived from the literature to establish 1) definite diabetes, 2) diabetes initially severe-requiring treatment with insulin, 3) remission of diabetes, and 4) exclusion of known causes of remitting diabetes.

**Results:**

10 out of 11 patients had an alternative explanation for their remission or a clear diagnosis was not identified. We identified a single patient with idiopathic remitting diabetes using these criteria. The patient was a white Caucasian female diagnosed aged 15 with symptoms of diabetes, laboratory glucose of 21.2 mmol/L and HbA1c 134 mmol/mol. Her BMI was 23.6 kg/m^2^. She was treated with basal bolus insulin but discontinued two years after diagnosis due to hypoglycaemia. 13 years post diagnosis, she had a normal oral glucose tolerance test during pregnancy (fasting glucose 4.5 mmol/L, 2 hr glucose 4.8 mmol/L) and an HbA1c of 30 mmol/mol. This patient does not appear to have Type 1 or Type 2 diabetes, and furthermore does not fit into current classifications of diabetes.

**Conclusions:**

Idiopathic remitting diabetes is rare but does exist. Strict clinical criteria are important to ensure patients have a robust clinical diagnosis. Identification of more patients with idiopathic remitting diabetes will enable further study of the clinical course of this syndrome. Applying these strict criteria will allow the identification of patients with remitting diabetes to assess its aetiology.

## Background

Diabetes is a heterogeneous disease that can occasionally remit. In the majority of remitting cases a cause can be identified. Examples include patients with Type 2 diabetes who lose weight through severe caloric restriction [1] or have had gastric bypass surgery [2], those with Type 1 diabetes who are in the honeymoon period [3], transient neonatal diabetes due to a methylation abnormality in chromosome 6q24 [4], and patients of African or Hispanic origin with ketosis-prone diabetes [5]. Rarely, patients initially assumed to have Type 1 diabetes have managed to maintain euglycaemia without treatment and have stopped using insulin for longer than would be expected of the honeymoon period [6]. This appears to be a unique form of diabetes that does not fit into the current classification of diabetes [7] and needs to be differentiated from other types.

If we could identify patients with idiopathic remitting diabetes, we would have an opportunity to study these patients to establish the cause of their diabetes and why it remits. At present there are no distinguishing clinical criteria to differentiate idiopathic remitting diabetes from other forms of diabetes.

Our aim was to identify patients with idiopathic remitting diabetes using robust clinical criteria. We systematically analysed patients referred to our centre with a diagnosis of “remitting diabetes” and applied literature-derived clinical criteria to exclude other forms of diabetes where remission can occur.

## Methods

### Subjects

We searched all referrals to our centre at the University of Exeter Medical School Clinical Research Facility (n > 15,000) between 2000 – 2012 for patients with a physician’s diagnosis of remitting diabetes, diagnosed between the ages of 0.5 – 35 years using the search terms remit, remit*, remis* in the database of referrals, excluding the 2 patients we have previously described [6].

### Data collection

We gathered longitudinal data from patients’ clinical records. We established patients’ clinical characteristics – age of diagnosis, ethnic origin, body mass index, mode of treatment, duration of treatment with insulin and how long they had been in remission. In addition we determined HbA1c and/or laboratory glucose at diagnosis and in remission, C-peptide or urinary C-peptide/creatinine ratio, and GAD/IA2 autoantibodies.

### Criteria

We then applied criteria to this cohort of 11 patients that defined patients with a definite diagnosis of diabetes, initial severe diabetes – requiring treatment with insulin, definite remission, and excluded other forms of diabetes that can remit in order to find patients with true idiopathic remitting diabetes. These criteria were derived from studies in the literature.

Diagnostic Criteria for Idiopathic Remitting Diabetes:

**Table 1:** Known types of remitting diabetes and the criteria applied to define them

**Recognised forms of remitting diabetes**	**Defining criteria**
Type 1 diabetes in the honeymoon period	GAD/IA2 positive
	Remission within four years of diagnosis
Type 2 diabetes with weight loss	Obesity at diagnosis with subsequent weight loss or bariatric surgery
Transient neonatal diabetes	Diabetes diagnosed under 6 months of age
Ketosis-prone atypical diabetes	Non-Caucasian
Stress induced hyperglycaemia or steroid-induced hyperglycaemia	Diagnosed at a time of extreme physiological stress e.g. acute illness
	Concurrent glucocorticoid use around the time of diagnosis

-Aged over 6 months or less than 35 years at diagnosis

-White Caucasian

-HbA1c laboratory glucose diagnostic of diabetes

-BMI less than 30 kg/m^2^

-Marked fasting hyperglycaemia (to exclude glucokinase-MODY) and requiring treatment with insulin from diagnosis

-4 consecutive years without insulin treatment and HbA1c in the normal range

-Other forms of remitting diabetes excluded (Table 1)

#### Definite diagnosis of diabetes

Patients should have a definite biochemical diagnosis of diabetes. We used WHO criteria based either on glucose (random glucose greater than 11.1 mmol/L with symptoms suggestive of diabetes, fasting glucose greater than 7.0 mmol/L, or a 2 hour glucose greater than 11.1 mmol/L) [8] or HbA1c ≥ 6.5% (48 mmol/mol) [9].

#### Diabetes initially severe – requiring treatment with insulin

Treatment is a marker of the severity of initial hyperglycaemia especially in young patients diagnosed before 35 years of age. We wanted there to be clear remission from initially marked hyperglycaemia. A patient requiring treatment with insulin from diagnosis is more likely to have marked hyperglycaemia and less likely to have Type 2 diabetes [10].

#### Definite remission

To have clear remission the patients should be able to stop all diabetes treatment and have normal glucose tolerance (HbA1c < 6.5%/48 mmol/mol). Type 1 diabetes is associated with a honeymoon period where insulin secretion is improved. This can lead to remission although most patients continue to take some insulin.

A period of remission of greater than 4 years is unusual for the honeymoon period. Previous studies have shown that 10% of patients with Type 1 diabetes are off insulin 12 months after initially commencing insulin [11]. Another study defined remission as an insulin dose of ≤ 0.3 units/kg/day with an HbA1c in the normal range, and found a prevalence of duration of remission of greater than 4 years of 2.5% in a sample of 362 patients [12].

We used a stricter definition of remission as defined by withdrawal of all treatment for diabetes for 4 years, with an HbA1c < 6.5% (48 mmol/mol) during this period.

#### Other forms of diabetes excluded

Our criteria excluded patients with a BMI above 30 kg/m^2^ who are those more likely to have Type 2 diabetes [13]. In obese patients any remission may be due to increased insulin sensitivity after weight loss [1]. Our patients were aged between 6 months and 35 years. An age over 6 months excludes transient neonatal diabetes [4] and an age at diagnosis of greater than 35 years is used by the Royal College of General Practitioners to identify patients more likely to have Type 2 diabetes [14]. Patients of non-European ancestry may have ketosis-prone diabetes, a form of diabetes that can remit [5,15,16], and so our criteria only includes patients of White European ancestry. We also excluded patients with stress hyperglycaemia [17] i.e. due to acute illness or those with hyperglycaemia secondary to corticosteroid use [18].

We thus defined idiopathic remitting diabetes as a non-obese, White European patient aged between 6 months and 35 years with a clear documented diagnosis of diabetes, treated with insulin from diagnosis, who had not required treatment for a period of 4 years or longer, with an HbA1c in the normal range when in remission, not on corticosteroids at the time of diagnosis.

## Results

### Most patients had an alternative reason for remission

**Figure 1:**
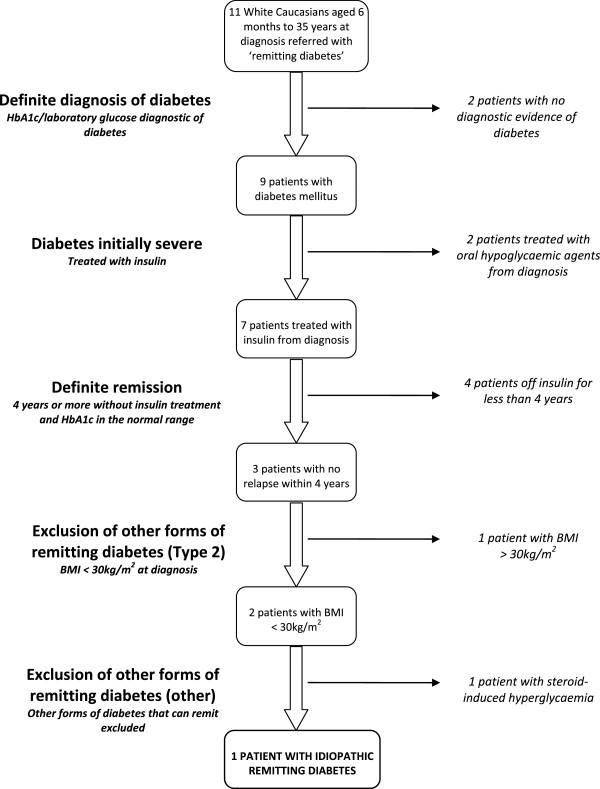
Flow chart showing referrals and the application of diagnostic criteria to diagnose idiopathic remitting diabetes.

We identified 11 patients referred with remitting diabetes. Our diagnostic criteria excluded 10 out of the 11 patients (Figure 1). All our patients were White European, aged less than 35 years at diagnosis, but none aged less than 6 months. 2 patients did not have a confirmed diagnosis of diabetes mellitus, one of the patients having only a capillary glucose value but no laboratory glucose or HbA1c. In total, 3 patients had a possible diagnosis of type 2 diabetes; 2 patients with a BMI above 30 kg/m^2^ and 1 patient treated with oral hypoglycaemic agents from diagnosis. 1 patient had a transient hyperglycaemia after a course of oral steroids.

### 1 patient had true idiopathic remitting diabetes

Application of our diagnostic criteria identified one patient with idiopathic remitting diabetes (Figure 1). This was a female patient diagnosed aged 15, after presenting with symptomatic diabetes and a period of rapid weight loss of approximately 12 – 15 kg over 2.5 months. Her laboratory glucose at diagnosis was 23 mmol/L and HbA1c was 14.4% (134 mmol/mol). Her BMI was 23.6 kg/m^2^. She was commenced on basal bolus insulin and remained on treatment for two years. She then stopped treatment due to hypoglycaemia; her HbA1c was 5.4% (36 mmol/mol). She had an oral glucose tolerance test aged 28 (fasting glucose 4.5 mmol/L, 2 hour glucose 4.8 mmol/L) during pregnancy. She has remained off treatment to date. There was no significant weight loss in the 15 years post-diagnosis making insulin resistance unlikely. GAD, IA2 and ZnT8 antibodies were negative, and she had preserved C-peptide with a UCPCR 9.98 nmol/mol (5th and 95th percentiles for non-diabetic controls 0.58 mmol/mol and 10.37 mmol/mol respectively) [19]. She has a family history of diabetes, with a maternal grandmother diagnosed with Type 2 diabetes, and a paternal uncle with Type 1 diabetes diagnosed in his teens. Neither parent has had diabetes. She has no siblings but her son, currently aged 3, does not have diabetes. Targeted next-generation sequencing of the 29 genes known to cause monogenic diabetes has not identified a mutation [20]. Her case does not fit in with any of the known causes of diabetes that remit. According to our criteria, the patient has idiopathic remitting diabetes, with her remission lasting even during pregnancy.

## Discussion

### Idiopathic remitting diabetes is rare

We have identified a cause for remission in 10 out of 11 patients with a physician’s diagnosis of remitting diabetes. Patients were either misdiagnosed or had another possible cause for their remitting hyperglycaemia. We identified a single patient with idiopathic remitting diabetes, on insulin for 2 years post diagnosis, and then remitting and remaining off treatment for the following 15 years, even during pregnancy. Idiopathic remitting diabetes is rare, as we have identified a single case from a database of >15,000 patients. Remitting diabetes of a similar pattern has been described by our group previously, affecting two young sibling boys of European ancestry [6]. We have not found any other cases in the literature.

### Strict criteria are necessary to identify patients who may have another cause for remission

Strict criteria have identified only 1 patient with idiopathic remitting diabetes. It may be that within our cohort of patients there are other patients with the same disease process who have not been identified due to the strict nature of our criteria. Inclusion of patients with a BMI above 30 kg/m^2^, for example, may yield a larger number of patients with idiopathic remitting diabetes, although as the average population BMI is increasing, there are likely to be more patients with Type 2 diabetes. However, it is only through using such strict criteria that we can be sure that there is not an alternative form of diabetes until more patients have been identified and studied to establish improved criteria, perhaps including other clinical characteristics, biomarkers or genetic testing.

### Case discussion

#### Remitting diabetes vs Type 1 diabetes

Our patient with idiopathic remitting diabetes does not have typical Type 1 diabetes. The prolonged length of time without insulin is not consistent with Type 1 diabetes in the honeymoon period. Whilst the honeymoon period is a common phenomenon in Type 1 diabetes, it is rare that it would continue for longer than 4 years and virtually all patients in a Swedish study looking at the length or remission after an initial diagnosis of Type 1 diabetes needed insulin therapy within this time period [12]. Our patient had preserved C-peptide secretion 13 years after her initial diagnosis of diabetes. This suggests that beta cell function was only temporarily disrupted. Previous work has demonstrated that patients this many years after a diagnosis of Type 1 diabetes are unlikely to be producing endogenous insulin in such quantities [21]. In addition, our patient has not developed diabetic ketoacidosis in the 15 years of her remission despite no insulin treatment. Negative GAD and IA2 antibodies also support that this is unlikely to be Type 1 diabetes [22]. The previous case report has also not identified insulin, islet cell or GAD autoantibodies in these patients [6].

#### Remitting diabetes vs Type 2 diabetes

This is not likely to be Type 2 diabetes in remission as our patient had a normal BMI at diagnosis in her teens. It is unusual to have an insulin requirement for 2 years after initial presentation before developing insulin independence in the absence of weight loss. Pregnancy is a period of heightened insulin resistance, and patients with risk factors may develop gestational diabetes [23]. Our patient has not relapsed, even after 13 years without insulin treatment and has even had normal glucose tolerance in pregnancy.

#### Rare forms of diabetes that can remit

The manner in which our patient’s diabetes progressed does not fit with glucokinase-MODY. Patients with glucokinase-MODY can be mistaken for having diabetes and can have an HbA1c in the diagnostic range [24]. These patients are often initially treated with insulin (perhaps due to their young age at diagnosis), then stop treatment due to perceived improvement in HbA1c and therefore appear to remit. HbA1c incorrectly diagnoses more patients who have glucokinase-MODY with diabetes compared with fasting glucose [24]. We have excluded glucokinase-MODY as our patient had markedly high initial glucose values which are now normal, evidenced also by the normal fasting glucose values our patient had in her OGTT in pregnancy (lower fasting glucose than seen in glucokinase-MODY [25]).

Ketosis-prone diabetes is another form of diabetes that can appear to remit, with patients developing episodes of acute hyperglycaemia and ketosis, requiring insulin and intermittent periods with no insulin-dependence [5]. Patients with remitting diabetes should be of white Caucasian ancestry to distinguish them from a possible diagnosis of ketosis-prone diabetes, which is well described in patients of non-Caucasian ancestry [16].

### Aetiology of idiopathic remitting diabetes

It is clear that more research is needed to establish the biology of this form of diabetes. Whilst it is difficult to establish an aetiology with so few cases described, it could be that it is due to a transient viral illness, or a burnt out autoimmune process which has been seen in other autoimmune conditions such as juvenile idiopathic arthritis [26] and membranous glomerulonephritis [27]. One theory postulates that Type 1 diabetes is a relapsing-remitting disease process with an interplay of autoreactive effector T cells and regulatory T cells dictating whether diabetes progresses or remits [28]. Parallels can also be drawn with multiple sclerosis, some patients having a single episode, others relapsing several times, and others with a primary progressive disease pattern [29]. An age of diagnosis greater than 6 months excludes transient neonatal diabetes, but this may be an area to look for comparisons in further research. Further exome sequencing of family members could uncover an as of yet undiscovered genetic cause, however given the absence of diabetes in the patient and her first degree relatives, this seems inappropriate.

### Limitations of the study

We have relied on data from clinical records which may be inaccurate or incomplete despite confirmation from hospital laboratories.

There are limitations to the way in which our criteria have been applied. Using strict criteria means that we may have excluded patients who may genuinely have idiopathic remitting diabetes, but given our data may be incomplete in some cases, using strict criteria is essential.

## Conclusion

It is unclear at present what the natural history, rate of complications, risk of relapse and aetiology is in patients with idiopathic remitting diabetes. More patients will need to be identified in order to study this further. In order to do this, we have found that identifying Remitting Diabetes of unknown aetiology requires the application of strict clinical criteria.

### Ethics statement

This study was not deemed to require REC approval under GAfREC guidelines as it involved research undertaken by staff within a care team using information previously collected in the course of care for their own patients or clients, which was pseudonymised in conducting the research.

## Competing interests

All authors’ declare that they have no competing interests.

## Authors’ contributions

All authors made substantial contributions to conception and design, acquisition of data, or analysis and interpretation of data. In addition, all authors were involved in drafting the article or revising it critically for important intellectual content and final approval of the version to be published. Any unpublished clinical data is held on our clinical database available to colleagues at the NIHR Exeter Clinical Research Facility only.

## Pre-publication history

The pre-publication history for this paper can be accessed here:

http://www.biomedcentral.com/1472-6823/14/45/prepub
